# Supplementary material for: An Integrative View of the Phyllosphere Mycobiome of Native Rubber Trees in the Brazilian Amazon
Source: J Fungi (Basel). 2022 Apr 6;8(4):373. doi: 10.3390/jof8040373 (PMC9025378; doi:10.3390/jof8040373)
Supplement: Supplementary file 1 [file jof-08-00373-s001.zip › Supplementary Material 1.pdf]

Supplementary Material 1: Bioinformatics pipelines

1. Amplicon Metagenomics

The output files (FASTQ format) of the amplicon metagenomic sequencing of each one of the samples comprise our raw primary data. The bioinformatics pipeline was developed an Operational System Ubuntu 16.04.5 LTS system. The programs used were: VSEARCH v2.9.1 (Rognes et al., 2016); BLAST v2.2.31+ (Camacho et al., 2009). Scripts in shell (McIlroy, 1987) and Python v3.6 (Martelli, 2006) programming languages were written to make some automatic tasks, such as merging samples or generating the abundance table. The reference database used for fungal taxonomic identification was UNITE v. 7.2 (Nilsson et al., 2010). The pipeline has the following steps, all of which using VSEARCH and BLASTn, as aforementioned: (i) quality and length filtering was done removing sequences smaller than 300 bp and default settings for quality filtering; (ii) dereplication; (iii) detection and removal of chimeric sequences using the UNITE database (uchime_reference_dataset_untrimmed.fasta) and de novo implementation); (iv) clustering sequences with similarity above 97%. All steps above we used VSEARCH software; (v) automatic taxonomic identification with BLASTn; and (vi) generation of the abundance table was built using python script.

References:

Camacho C, Coulouris G, Avagyan V, Ma N, Papadopoulos J, Bealer K, Madden TL. 2009.BLAST+: architecture and applications. *BMC Bioinformatics 10(1):1–9* DOI 10.1186/1471-2105-10-421.

Martelli A. 2006. Python in a nutshell: A desktop quick reference. *New York: O'Reilly Media, Inc., 738.*

McIlroy MD. 1987. A research UNIX reader: annotated excerpts from the programmer's manual, 1971–1986. *Murray Hill: AT&T Bell Laboratories.*

Nilsson, R.H., Larsson, K.H., Taylor, A.F.S., Bengtsson-Palme, J., Jeppesen, T.S., Schigel, D., Kennedy, P., Picard, K., Glöckner, F.O., Tedersoo, L. and Saar, I., 2019. The UNITE database for molecular identification of fungi: handling dark taxa and parallel taxonomic classifications. *Nucleic acids research*, *47*(D1), pp.D259-D264.

Rognes T, Flouri T, Nichols B, Quince C, Mah F. 2016. VSEARCH: a versatile open source tool for metagenomics. *PeerJ 2016(10):22* DOI 10.7717/peerj.2584.

Pipeline for metagenomic ITS
This pipeline was elaborated and run on an Ubuntu 16.04.5 LTS system
Programs
- Vsearch v2.9.1
wget https://github.com/torognes/vsearch/archive/v2.9.1.tar.gz
- Blast v2.2.31+
sudo apt-get install ncbi-blast+
- Python
sudo apt-get install python
- Perl
sudo apt-get install perl
FASTA file processing
for R in *_R1.fastq ; do
vsearch --fastq_mergepairs ${R} --reverse ${R/_R1/_R2} --fastqout merged${arq}.fastq
**Merge paired-end sequence reads into one sequence.
vsearch --fastq_filter merged${arq}.fastq --fastq_maxee 0.5 --fastq_minlen 300 --eeout --fasta_width 0 --fastaout filtered${arq}.fasta
**Shorten and/or filter the sequences in the given FASTQ file and output the remaining sequences to the FASTQ file specified
vsearch --derep_fulllength filtered${arq}.fasta --minuniquesize 2 --strand plus --sizeout --uc dereplicated${arq}.uc --relabel sample_${arq}id_ --fasta_width 0 --output dereplicated${arq}.fasta
**Merge strictly identical sequences contained in filename. Identical sequences are defined as having the same length and the same string of nucleotides (case insensitive, T and U are considered the same).
end
Merge all samples
python3 catDerep_JF.py
Precluster before chimera detection
vsearch --cluster_size all_dereplicatedS.fasta --id 0.97 --strand plus --sizein --sizeout --fasta_width 0 --uc precluster.uc --centroids precluster.fasta
**Clusterize the fasta sequences in filename, automatically perform a sorting by decreasing sequence abundance beforehand.
Detect chimeras present in the fasta-formatted (denovo)
vsearch --uchime_denovo precluster.fasta --sizein --sizeout --fasta_width 0 --nonchimeras denovo_nonchimeras.fasta
**Without external references (i.e. de novo). Automatically sort the sequences in filename by decreasing abundance beforehand (see the sorting section for details). Multithreading is not supported.
Download UNITE database
https://unite.ut.ee/repository.php
Detect chimeras present in the fasta-formatted (reference)
vsearch --uchime_ref denovo_nonchimeras.fasta --db uchime_reference_dataset_untrimmed_28.06.2017.fasta --sizein --sizeout --fasta_width 0 --nonchimeras ref_nonchimeras.fasta
**Detect chimeras present in the fasta-formatted filename by comparing them with reference sequences (option --db). Multithreading is supported.
Extract all non-chimeric, non-singleton sequences, dereplicated
perl map.pl all_dereplicatedS.fasta precluster.uc ref_nonchimeras.fasta >

nonchimeras_derep.fasta
Extract all non-chimeric, non-singleton sequences in each sample
perl map.pl dereplicatedS.fasta ll_dereplicatedS.uc nonchimeras_derep.fasta > all_nonchimeras_derep.fasta
Clustering
vsearch --cluster_size all_nonchimeras_derep.fasta --id 0.97 --sizein --sizeout --fasta_width 0 --relabel otu_ --uc otus.uc –centroids otus.fasta
**Clusterize the fasta sequences in filename, automatically perform a sorting by decreasing sequence abundance beforehand.
Identification OTUs usando BLAST
blastn --db base -qcov_hsp_perc 90.0 -perc_identity 97.0 -query otus.fasta -outfmt '6 qseqid stitle pident qcovhsp' -out taxonomy.blast
**BLASTn was used to compare the reads with the in house reference fungal BLAST database. Only sequences with at least 97% similarity and at least 90% coverage were identified. OTUs with ≥ 98,5% of similarity was assigned to species level and similarities between 97 and 98,5% was assigned to genus level.
Generating the Abundances Table
python abund.py
**We used a script written in python to parse the identification output and generate a table in CSV (Comma-Separated Values) format
Some flags used:
--fastq_minlen~positive integer
With the --fastq_filter and --fastq_mergepairs commands, discard sequences with less than the specified number of bases (default 1).
--fastq_maxee~real
With the --fastq_filter and --fastq_mergepairs commands, discard sequences with more than the specified number of expected errors.
--fasta_width~positive integer
Fasta files produced by vsearch are wrapped (sequences are written on lines of integer nucleotides, 80 by default). Set that value to 0 to eliminate the wrapping.
--minuniquesize~positive integer
Discard sequences with an abundance value smaller than integer.
--relabel string
Please see the description of the same option under Chimera detection for details.
--sizein
Take into account the abundance annotations present in the input fasta file (search for the pattern "[>;]size=integer[;]" in sequence headers).
--sizeout
Add abundance annotations to the output fasta file (add the pattern ";size=integer;" to sequence headers). If --sizein is specified, each unique sequence receives a new abundance value corresponding to its total abundance (sum of the abundances of its occurrences). If --sizein is not specified, input abundances are set to 1, and each unique sequence receives a new abundance value corresponding to its number of occurrences in the input file.

Rules for identification of fungal OTUs:

```
if (similarity >= 97):
   return genus                   #genus
elif (similarity < 97) and (similarity >= 95):
   return family                  #family
elif (similarity < 95) and (similarity >= 90):
   return order                   #order
elif (similarity < 90) and (similarity >= 85):
   return class                   #class
elif (similarity < 85) and (similarity >= 80):
   return phylo                   #phylum
elif (dado_id < 80):
   return kingdom                 #kingdom
end
```
